# Tropical and montane *Apis cerana* show distinct dance–distance calibration curves

**DOI:** 10.1242/jeb.247510

**Published:** 2024-07-03

**Authors:** Bharath Kumar A. K., Ebi Antony George, Axel Brockmann

**Affiliations:** ^1^ National Centre for Biological Sciences - Tata Institute of Fundamental Research, Bengaluru 560065, India; ^2^ Department of Apiculture, University of Agricultural Sciences - GKVK, Bengaluru 560065, India; ^3^ Department of Ecology and Evolution, Biophore, University of Lausanne, 1015 Lausanne, Switzerland

**Keywords:** Honey bees, Waggle dance, Communication, Geographic variation, Odometer

## Abstract

Social bees have evolved sophisticated communication systems to recruit nestmates to newly found food sources. As foraging ranges can vary from a few hundred meters to several kilometers depending on the environment or season, populations of social bee species living in different climate zones likely show specific adaptations in their recruitment communication. Accordingly, studies in the western honey bee, *Apis mellifera*, demonstrated that temperate populations exhibit shallower dance-calibration curves compared with tropical populations. Here, we report the first comparison of calibration curves for three Indian *Apis cerana* lineages: the tropical *Apis indica*, and the two montane Himalayan populations *Apis cerana cerana* (Himachal Pradesh) and *Apis cerana kashmirensis* (Jammu and Kashmir). We found that the colonies of the two montane *A. cerana* populations show dance–distance calibration curves with significantly shallower slopes than those of the tropical *A. indica*. Next, we transferred *A. c. cerana* colonies to Bangalore (∼ 2600 km away) to obtain calibration curves in the same location as *A. indica*. The common garden experiment confirmed this difference in slopes, implying that the lineages exhibit genetically fixed differences in dance–distance coding. However, the slopes of the calibration curves of the transferred *A. c. cerana* colonies were also significantly higher than those of the colonies tested in their original habitat, indicating an important effect of the environment. The differences in dance–distance coding between temperate and tropical *A. cerana* lineages resemble those described for *Apis mellifera*, suggesting that populations of both species independently evolved similar adaptations.

## Teaser

**Summary:** Dance–distance calibration curves of the montane *Apis cerana cerana* and *Apis cerana kashmirensis* are shallower than those of the tropical *Apis indica*, highlighting geographic variation in waggle dance behavior within *A. cerana* lineages.

## INTRODUCTION

Honey bees use the symbolic dance communication to indicate flight direction and distance of food sources to nestmates in the hive (von Frisch, 1967; Riley et al., 2005). This behavior was initially observed in the European honey bee *Apis mellifera carnica* and the question was raised early on whether populations and species of honey bees differ in dance communication similar to dialects in human and bird vocal communication (‘dance dialects’; see von Frisch, 1948). Today, there is good evidence that honey bee species and populations of *A. mellifera* differ in several aspects of distance coding and that some of those are heritable (Boch, 1957; Dyer and Seeley, 1991; Johnson et al., 2002; Kohl et al., 2020; Lindauer, 1956; Rinderer and Beaman, 1995; Schneider, 1989). The adaptive tuning hypothesis proposed that the rate of change in dance duration with distance is negatively correlated with the mean foraging range of a population or species (Dyer and Seeley, 1991; Gould, 1982; Kohl et al., 2020; Punchihewa et al., 1985). Consequently, it is assumed that populations or species with larger foraging ranges have dance–distance calibration curves with shallower slopes, which reduces the accuracy with which they communicate small changes in flight distances.

The modern honey bee species evolved in the tropics of Asia and only populations of the two cavity-nesting species, *A. mellifera* and *Apis cerana*, extended their distribution ranges into temperate climate zones (Ji et al., 2020; Ruttner, 1988; Smith, 2020). Compared with the tropics, temperate regions exhibit stronger seasonal variation in food abundance and long periods of food shortage during winter. Colonies of temperate populations are generally larger in worker number and build up substantial food stores (Ruttner, 1988; Seeley and Visscher, 1985). Higher food demands and lower food availability can be countered by enlarging colony foraging ranges (Beekman and Ratnieks, 2000; Couvillon et al., 2014; Grüter and Hayes, 2022; Young et al., 2021). Accordingly, temperate populations of both species show shallower dance–distance calibration curves associated with larger foraging ranges (Boch, 1957; Hu et al., 2023; Kohl et al., 2020; Punchihewa et al., 1985; Sasaki et al., 1993; Schneider, 1989; Su et al., 2008). However, only Boch (1957) and Kohl and colleagues (2020) compared the dance behavior of different *A. mellifera* populations in common garden experiments to exclude any possible environmental effects. This is crucial as it was shown that honey bees use optical flow to measure flight distances and include this information in their dance communication (Esch et al., 2001; Srinivasan et al., 2000; Tautz et al., 2004).

**Fig. 1.**
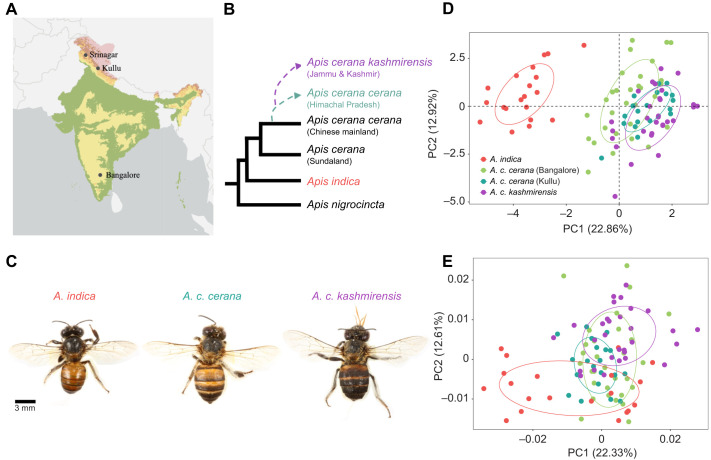
**Tropical and montane lineages of *Apis cerana* differ in their morphological characteristics.** (A) Map of India with experimental locations highlighted. (B) Phylogeny of the *Apis cerana* group (modified after Qiu et al., 2023; Smith, 2020). (C) Photographs of workers of the three lineages. (D) Principal components analysis (PCA) plot of all morphometric characteristics. (E) PCA plot of geometric morphometric wing-shape characteristics. In D and E, the filled circles represent individual workers while the large ellipses represent a normal distribution at the 0.5 level around the points for each lineage in each location. The ellipses are colored by lineage and location, with red for *Apis indica*, green for *Apis cerena cerana* in Bangalore, teal for *A. c. cerana* in Kullu and purple for *Apis cerena kashmirensis.*

In India, there are at least three *A. cerana* lineages according to recent morphological and genomic studies: *Apis indica* (originally called *A. cerana indica* or ‘yellow Indian’ honey bee), *Apis cerana* (=*A. cerana cerana* or ‘black Indian’ honey bee) and *Apis cerana kashmirensis* (see Fig. 1; Smith, 2020; Abrol, 2013; Hepburn et al., 2001; Qiu et al., 2023; Su et al., 2023). *Apis indica* stems from a population that invaded India before the last Ice Age and survived in the southern parts, whereas the *A. cerana* lineage descended from a second colonization (Smith, 2020). Although recent molecular studies support earlier reports that *A. cerana* and *A. indica* might represent separate species, hybrid colonies of black and yellow honey bees are common in the tropical region of southern India (Lo et al., 2010; Oldroyd et al., 2006; Otis and Smith, 2021; Smith, 2020; Su et al., 2023; Viraktamath et al., 2013). Thus, it is unclear whether *A. indica* is a distinct species or an *A. cerana* population at a late state of divergence and we decided to use the term ‘lineage’ to highlight the difference of this bee compared with the other two *A. cerana* populations. The *A. cerana* of the second invasion diverged into several populations adapting to different habitats on the Indian subcontinent. There is good evidence that there are two northern montane populations: *A. c. kashmirensis* in the Jammu and Kashmir region and *A. c. cerana* in the central and eastern Himalayas (Hepburn et al., 2001; Qiu et al., 2023; Su et al., 2023; Viraktamath et al., 2013). These montane bees experience shorter flowering seasons and snowfall for at least a few weeks, similar to temperate *A. cerana* and *A. mellifera* populations (Abrol, 2013; Ahmad, 2023; Ji et al., 2020). Both environmental conditions, as discussed above, suggest larger foraging ranges and shallower dance–distance curves compared with the tropical *A. indica*. According to the differences in environmental conditions and the prediction of the adaptive tuning hypothesis, the tropical *A. indica* should show a steeper slope of the dance–distance calibration curve than the Himalayan *A. cerana* populations. On the basis of these ideas, we determined the dance–distance calibration curves of *A. c. kashmirensis* (Kashmir), *A. c. cerana* (Himachal Pradesh) and *A. indica* (Bangalore). First, we compared colonies of the two Himalayan lineages, *A. c. kashmirensis* and *A. c. cerana*, within their natural distribution range. Then, we transported three *A. c. cerana* colonies from Himachal Pradesh to the tropical region of Bangalore (∼2600 km distance) and performed a common garden experiment testing them in the same location where we had tested colonies of the *A. indica* lineage. We found that colonies of the two Himalayan *A. cerana* populations exhibit dance–distance calibration curves with significantly shallower slopes than those of *A. indica* colonies, and this difference persisted in the common garden experiment. However, the calibration curves of the *A. c. cerana* colonies tested in Bangalore were steeper than those of colonies tested in the Himalayas, indicating that environmental differences also influence calibration curves. Our findings indicate that temperate and tropical *A. cerana* lineages exhibit adaptations in dance–distance coding similar to temperate and tropical *A. mellifera* populations.

## MATERIALS AND METHODS

### Honey bee colonies and location of experiments

Colonies of the three *A. cerana* lineages were bought from local beekeepers within their natural ranges: *A. indica* (Department of Apiculture, University of Agricultural Sciences, Bangalore, UAS-GKVK), *A. c. cerana* (Deen Dayal Bee Farm, Kullu, Himachal Pradesh), *A. c. kashmirensis* (Imtiyaz Qureshi Bee Farm, Srinagar, Jammu and Kashmir). In the Himalayas, the *A. c. cerana* and *A. c. kashmirensis* colonies were tested at field sites around 20 km away from the farms of the beekeepers. Three colonies of *A. c. cerana* (obtained from Deen Dayal Bee Farm) were transported by car over a distance of around 2600 km from Kullu to Bangalore at the end of October 2022. They were initially kept on the campus of the National Centre for Biological Sciences. Then, the experiments were started at the end of December 2022 on the campus of the neighboring University of Agricultural Sciences, UAS-GKVK. This schedule was chosen to allow the transported colonies to settle and adapt to the new location and to reduce the likelihood of absconding. The colonies were observed once a week after they were brought to Bangalore, and although they produced drone brood cells, they never made any queen cells. Thus, these colonies did not undergo any emergency supersedure or mating of a newly reared queen with drones from other colonies.

### Morphometric measurements

We performed morphometric analyses to confirm the lineage status of the colonies used in this study. From each colony, 10 foragers were collected at the end of the experiments described below for standard and geometric morphometric analysis. Photographs of the thorax and wings were taken with a stereomicroscope (Leica M125 C; camera: Leica MC 190 HD; software: LAS V4.12). For the photographs, the forewings and hindwings were mounted on a glass slide using a coverslip. Measurements were performed on those photographs using Fiji software (Schindelin et al., 2012). For the standard morphometric analysis, we measured: the intertegular distance (Cane, 1987), hamuli number, cubital index (described in Qiu et al., 2023), and 14 wing characters: forewing length, forewing width (wfw), length of cubital vein 1 and 2 (Cub1, Cub2), and 10 wing vein angles (A4, B4, D7, E9, G18, J16, K19, L13, N23, 026) (Ruttner, 1988). For the geometric morphometric analysis, 20 forewing landmarks were used (Ji et al., 2020). Images of the forewings were first digitized using tpsDig v2.0 and tpsUtil v1.40 (https://sbmorphometrics.org/) and then we obtained the two-dimensional landmark coordinates for further analysis.

### Experimental procedure

We followed the same experimental protocol for all colonies. Table 1 comprises information on colonies, dates, location of experiments and the general flowering conditions during the time the experiments were performed.

**Table 1.**
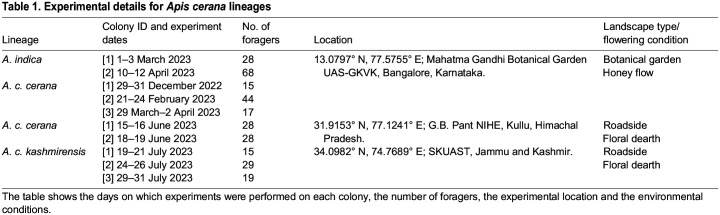
Experimental details for *Apis cerana* lineages

Prior to the experiments, colonies were transferred to observation hives during the evening hours after sunset. The observation hives were custom built similar to single hive boxes in which the frames (five for each tested colony) were arranged horizontally, and only one comb was exposed to the observation window (Kohl et al., 2020). Colonies were left undisturbed for a day before we started training foragers. To train foragers, a piece of honeycomb filled with 2 mol l^−1^ sugar syrup was first placed at the entrance of the hive. When a group of about 20 foragers continuously collected sugar syrup, the comb was moved to a gravity feeder containing 2 mol l^−1^ sugar syrup kept on a stand 10 m from the hive entrance. The piece of comb was removed when all the bees started collecting from the gravity feeder. Bees were individually color marked on the thorax and abdomen (Uni POSCA Paint Markers, Uni Mitsubishi Pencil, Tokyo, Japan). During the experiments, the feeder setup was gradually moved from one feeder distance to the next at a rate of approximately 10 m per 5 min. The first feeder station at which dances were recorded was 50 m from the hive. Feeder stations were separated by 50 m and the bees were trained to a maximum distance of 500 m from the hive in Bangalore and Kashmir, and to a distance of 400 m in Kullu (with the exception of colony 1 of *A. c. cerana* in Bangalore, which could be trained only to 300 m). Waggle dances were recorded using the Sony FDR-AX53 Handycam (Sony Corporation, Tokyo, Japan) at 60 frames s^−1^. At each feeder station, dances were recorded for 1 h. Unmarked foragers which arrived at the feeder were collected, kept in boxes during the experiment and released at the end of the day. Experimental duration per colony ranged from 3 to 5 days. Each following day, the experiment was resumed at the last feeder position of the previous day.

### Measurement of waggle phases

Analysis of the dance recordings was done using VitualDub2 software (http://virtualdub2.com/). The waggle phase duration was calculated by counting the number of frames between the start and end of the waggle phase and multiplying it with the frame rate of the recording. We defined the start frame as the first frame in which the bee started to move its abdomen sideways and the end frame as the frame in which the bee stopped moving its abdomen back and forth. All the analyses on the waggle dance were performed on the mean waggle phase duration per dance and not on individual waggle phases. Thus, for each distance, we had multiple dances from multiple individuals in a given colony.

### Statistics

We used generalized linear mixed models (GLMMs) for our statistical comparisons on the waggle dance. For the morphometric analysis, we used dimensionality reduction analysis to identify differences between the four groups as well as linear mixed models for comparing specific traits.

#### Morphometric analysis

Principal component analysis (PCA) based on all 17 measured standard morphological traits was used to identify how individual foragers clustered. For the geometric morphometric analysis, a PCA was performed using the size-corrected shape data in MorphoJ v.1.08.01 (Klingenberg, 2011) to compare differences amongst the lineages. In both PCAs, we plotted the distribution of individual data points on the first and second principal components, colored the data points per lineage and location, and visually examined how the groups clustered.

#### Lineage differences in dance–distance calibration curves

To compare the dance dialects between the different lineages, we fitted linear mixed-effects models (LMMs) with the mean waggle phase duration as the response variable, and an interaction between distance (a continuous variable) and lineage (a categorical variable with four levels: *A. indica*, *A. c. cerana* Bangalore, *A. c. cerana* Kullu, and *A. c. kashmirensis*) as the predictor variable. For the random effect structure, we first fitted a random slopes model with an effect of individuals nested within colonies on the slope linking mean waggle phase duration and distance. However, because of convergence issues with this model, we then fitted a random intercept model with an effect of colony on the intercept. After model validation, we used the random intercept model for inference and estimated marginal trends for the slopes for each level of the lineage. We compared significant differences between each pair of slopes using the Tukey method for *P*-value adjustment.

For comparison of the dance dialects, we also fitted non-linear mixed-effects models (NLMM). We did this as previous work has shown that non-linear curves often fit the waggle dance duration–distance relationship better (George et al., 2021; Kohl and Rutschmann, 2021). We fitted a logarithmic curve as described previously (George et al., 2021). Finally, we also fitted a LMM with a random effect of individual bee ID on the intercept. In this model, all the remaining predictors remained the same as in the previous LMM. We then compared between the three models (LMM with colony intercept as the random factor, LMM with individual bee intercept as the random factor and NLMM with colony slopes as the random factor) using multiple metrics including the Akaike information criterion (AIC), the Akaike information criterion for small samples (AICc) and the Bayesian information criterion (BIC). The LMM with colony intercept was the best-fitting model amongst the three and hence we provide the results of this model in the paper. Results of the mode comparison and the second best performing model, the NLMM, are provided in Tables S1 and S4. All three model assumptions were validated, and all three models gave similar results with respect to our questions of interest.

#### Colony variation

To look at colony variation in slopes within lineages, we used LMMs. We first created a dummy variable ‘lineage_colony’, which was an interaction between the lineage categorical variable of four levels described above and the colony ID (categorical variable of maximum three levels). We then fitted the model with the mean waggle phase duration as the response variable, and an interaction between distance (a continuous variable) and lineage_colony (a categorical variable with 10 levels: two colonies of *A. indica*, three colonies of *A. c. cerana* Bangalore, two colonies of *A. c. cerana* Kullu and three colonies of *A. c. kashmirensis*) as the predictor variable. We used this categorical variable to minimize the number of models we ran (four different models if we looked at each lineage separately instead of one model). For the random effect structure, we first fitted a random slopes model with an effect of individual bee on the slope linking mean waggle phase duration and distance. However, because of convergence issues with this model, we fitted a random intercept model with an effect of individual bee on the intercept. After model validation, we used this model for inference and estimated marginal trends for the slopes for each level of the lineage. We compared significant differences between each pair of slopes using the Tukey method for *P*-value adjustment. We report differences between colonies of the same lineage in the Results.

#### Inter-individual variation

To look at individual variation in the slope of the relationship between waggle phase duration and distance, we first shortlisted individuals which performed at least one dance at three unique distances. This gave us a final dataset of 81 individuals across 10 colonies (mean±s.d. 8.1±5.17, range 1–15 individuals; Fig. S1). We then created a dummy categorical variable ‘lineage_colony_beeID’ as described above for the colony comparison incorporating lineage, colony and bee ID (our bee IDs were based on color combinations and hence repeated across colonies and lineages). We then fitted a LMM with mean waggle phase duration as the response variable, and an interaction between distance (a continuous variable) and lineage_colony_beeID (a categorical variable with 81 levels for each individual) as the predictor variable. For the random effect structure, we first fitted a random slopes model with an effect of lineage_colony (described above) on the slope linking mean waggle phase duration and distance. However, because of convergence issues with this model and a random intercept model with an effect of lineage_colony on the intercept, we finally fitted a simple linear model without any random effects. After model validation, we used this model for inference and estimated slope values for 81 individuals. We further shortlisted this set of slope values to remove four individuals which had negative estimated slopes (due to either low dances in one distance, or very few data points overall).

Next, to compare individual variation across lineages, we obtained the coefficient of variation of individual slopes for each colony within each lineage. As we had only one individual for *A. c. cerana* Bangalore colony 3, we could not estimate the coefficient of variation for this colony. We then fitted a linear model with the coefficient of variation in individual slopes as the response and the lineage (categorical variable of four levels) as the predictor. After model validation, we used this model for inference and estimated marginal means for each level of the lineage. We compared significant differences between each pair of estimated marginal means using the Tukey method for *P*-value adjustment.

We used R v4.3.2 (http://www.R-project.org/) along with RStudio IDE (https://posit.co/products/open-source/rstudio/) to perform all the statistical analyses except for the PCA of geometric morphometric wing-shape characteristics. GLMMs were built using the lme4 package in R (Bates et al., 2015), while we used the nlme (https://CRAN.R-project.org/package=nlme) and aomisc package (https://www.statforbiology.com/) to build the non-linear mixed effects models. Model validation was performed using DHARMa (https://CRAN.R-project.org/package=DHARMa) and performance (Lüdecke et al., 2021) packages, while marginal means and contrasts were obtained using the emmeans (https://CRAN.R-project.org/package=emmeans) and modelbased (https://CRAN.R-project.org/package=modelbased) packages. The PCA was performed using the factoextra package (https://CRAN.R-project.org/package=factoextra). For the PCA of geometric morphometric wing-shape characteristics we used the MorphoJ v.1.08.01 (Klingenberg, 2011) software. All the plots were created in R using ggplot2 (Wickham, 2016) and cowplot (https://CRAN.R-project.org/package=cowplot) packages.

## RESULTS

### Morphometric analysis

The morphological analyses confirmed the phylogenetic lineage of the colonies used in our experiments. The PCA including all the standard morphological characters (i.e. size-dependent and -independent characters) clearly separated the *A. indica* specimens from those of the two montane northern lineages *A. c. cerana* and *A. c. kashmirensis* (Fig. 1D). PC1, which accounted for 22.9% of the total variation, was mainly influenced by intertegular distance, width of forewing, length of forewing, length of cubital vein 1 and wing vein angle K19 (loading values of 0.43, 0.42, 0.41, 0.35 and 0.35, respectively), while PC2, which accounted for 12.9% of the total variation, was influenced by length of cubital vein 2, cubital index and wing vein angles A4 and D7 (loading values of −0.43, −0.41, −0.41 and −0.38, respectively). PC3, which accounted for 11.6% of the total variation, was influenced wing vein angles N23, J10, B4 and J16 (loading values of 0.46, 0.41, −0.38 and −0.38, respectively. The eigenvalues of PC1, PC2 and PC3 were 4.11, 2.32 and 2.08, respectively, and the loading values of all the other variables on each PC are given in Table S2.

The PCA of the size-corrected geometric morphometric landmarks of the forewings showed similar results to the PCA on the standard morphological characteristics (Fig. 1E). There was complete overlap between the montane lineages, while the overlap of these lineages with *A. indica* was minimal. PC1 accounted for 22.33% of the total variation, while PC2 accounted for 12.61% and PC3 accounted for 9.61%. The eigenvalues of PC1, PC2 and PC3 were, respectively, 0.00099, 0.00056 and 0.00042, and the loading values of all the other variables on each PC are given in Table S3.

### Lineage differences in dance–distance calibration curves

**Fig. 2.**
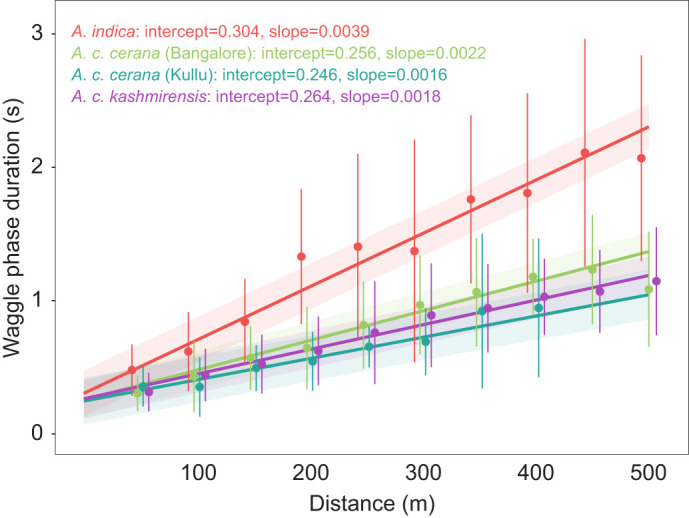
**The dance–distance calibration curve of the tropical *A.*** ***indica*** **exhibits a steeper slope than that of the two montane *A. cerana* lineages.** Circles and error bars represent mean and standard deviation of waggle phase duration for each distance from 15–68 foragers per colony and 2–3 colonies per lineage in each location. The line represents the estimated trend line obtained from the linear mixed effects model and the shaded region represents the 95% confidence interval around the estimated trend. The intercept and slope values of the estimated trend are given. Circles, error bars, lines and shaded region are colored per lineage and location, with red for *A. indica*, green for *A. c. cerana* in Bangalore, teal for *A. c. cerana* in Kullu and purple for *A. c. kashmirensis*.

The slopes of mean dance–distance calibration curves based on data from 15–68 foragers per colony and two to three colonies per lineage were significantly different between tropical and montane *A. cerana* lineages (intercept and slope values provided in Fig. 2).

The calibration curves of the tropical *A. indica* showed the steepest slope, significantly higher than those of the calibration curves of *A. c. cerana* and *A. c. kashmirensis* (*A. indica* versus *A. c. cerana* in Kullu: difference estimate=0.0024, *t*-ratio=15.09, *P*<0.0001; *A. indica* versus *A. c. kashmirensis*: difference estimate=0.0021, *t*-ratio=19.55, *P*<0.0001). Moreover, the difference in the slopes between *A. indica* and *A. c. cerana* did not change in the common garden experiment performed in Bangalore (*A. indica* versus *A. c. cerana* in Bangalore: difference estimate=0.0018, *t*-ratio=16.08, *P*<0.0001). The slopes of the calibration curves of the two Himalayan lineages *A. c. cerana* and *A. c. kashmirensis* did not differ when tested in the montane regions (*A. c. cerana* in Kullu versus *A. c. kashmirensis*: difference estimate=−0.0003, *t*-ratio=−1.52, *P*=0.43). However, *A. c. cerana* colonies transferred from Kullu to Bangalore exhibited calibration curves with significantly higher slopes than the colonies from the same population tested in Kullu (*A. c. cerana* in Bangalore versus *A. c. cerana* in Kullu: difference estimate=0.0006, *t*-ratio=3.73, *P*=0.001).

### Colony variation

**Fig. 3.**
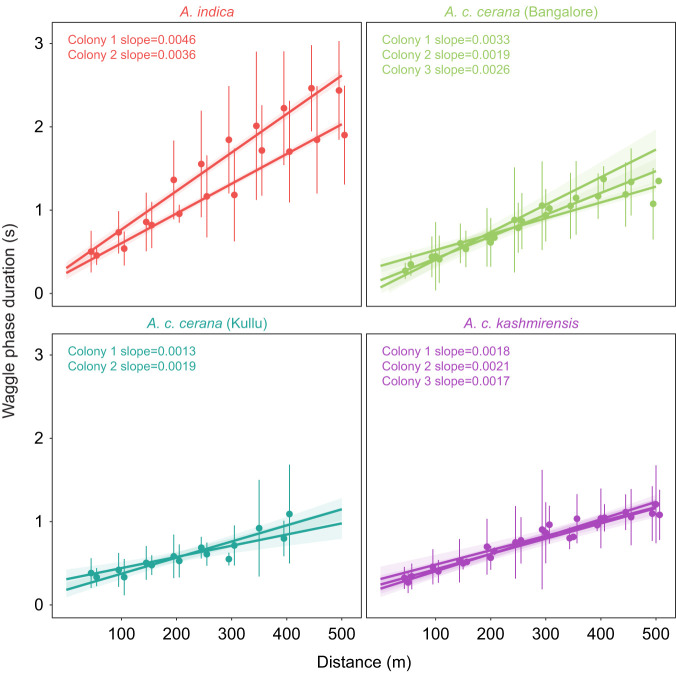
**Dance–distance calibration curve of the colonies varies by lineage.** Each subplot represents all the colonies tested for one lineage in one location with 15–68 foragers per colony. The circles and error bars represent mean and standard deviation of waggle phase duration for each distance. The line represents the estimated trend line obtained from the linear mixed effects model and the shaded region represents the 95% confidence interval around the estimated trend. The slope values of the estimated trend for each colony are given. Circles, error bars, lines and shaded region are colored per lineage and location, with red for *A. indica*, green for *A. c. cerana* in Bangalore, teal for *A. c. cerana* in Kullu and purple for *A. c. kashmirensis*.

Colony variation in the slopes of the calibration curves differed by lineage (slope values provided in Fig. 3). The two *A. indica* colonies significantly differed in the slopes of the dance–distance calibration curves (colony 1 versus colony 2: difference estimate=0.0011, *t*-ratio=7.17, *P*<0.0001), whereas the two colonies of *A. c. cerana* as well as the three colonies of *A. c. kashmirensis* tested in their home range did not show significant differences (*A. c. cerana* in Kullu: colony 1 versus colony 2: difference estimate=−0.0006, *t*-ratio=−1.76, *P*=0.762; *A. c. kashmirensis*: colony 1 versus 2: difference estimate=−0.0003, *t*-ratio=−1.13, *P*=0.982; colony 1 versus 3: difference estimate=0.0001, *t*-ratio=0.43, *P*=0.999; colony 2 versus 3: difference estimate=0.0004, *t*-ratio=1.50, *P*=0.892). In contrast to the experiments in the Himalayas, the three *A. c. cerana* colonies transported to and tested in Bangalore showed some degree of variation in the slopes of the calibration curves. Colonies 1 and 2 significantly differed in their slopes, but the slope for colony 3 did not differ from that of colony 1 or colony 2 (colony 1 versus 2: difference estimate=0.0014, *t*-ratio=3.92, *P*=0.004; colony 1 versus 3: difference estimate=0.0007, *t*-ratio=1.57, *P*=0.862; colony 2 versus 3: difference estimate=−0.0007, *t*-ratio=−2.44, *P*=0.306).

### Inter-individual variation

**Fig. 4.**
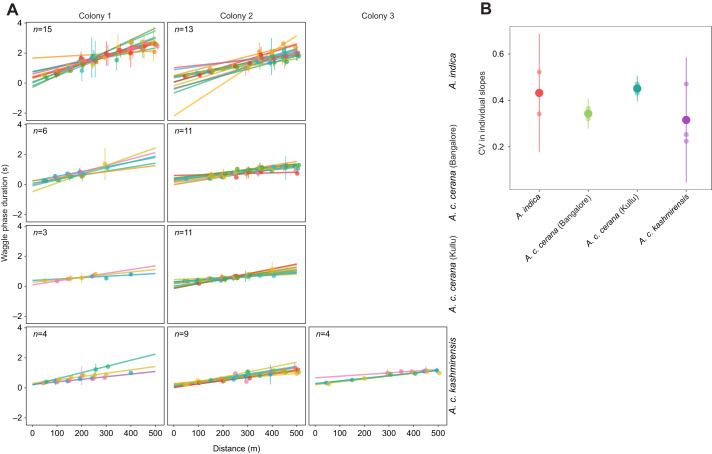
**Variation in dance–distance calibration curve of individuals is similar across lineages.** Each subplot represents the dance–distance calibration curve for all shortlisted individuals in one colony for each lineage. Circles and error bars represent the mean and standard deviation of waggle phase duration per individual for each distance and the line represents the estimated trend line obtained from the linear model. Circles, error bars and lines are colored based on individual ID. (B) Small circles represent the coefficient of variation of individual slopes for one colony with larger circles and error bars representing the mean and standard deviation of the coefficient of variation across lineages at each location. Circles and error bars are colored per lineage and location, with red for *A. indica* (15 and 13 individuals for colony 1 and 2), green for *A. c. cerana* in Bangalore (6 and 11 individuals for colony 1 and 2), teal for *A. c. cerana* in Kullu (3 and 11 individuals for colony 1 and 2) and purple for *A. c. kashmirensis* (4, 9 and 4 individuals for colony 1, 2 and 3).

Individual foragers of the different lineages showed considerable variation in the slopes of the dance–distance calibration curves (Fig. 4A; individual slope values provided in Table S4): *A. indica* (minimum slope=0.0009, maximum slope=0.0106), *A. c. cerana* in Bangalore (minimum slope=0.0004, maximum slope=0.0058), *A. c. cerana* in Kullu (minimum slope=0.0007, maximum slope=0.0033) and *A. c. kashmirensis* (minimum slope=0.0011, maximum slope=0.0041). However, the coefficient of variation in the slopes of individuals did not differ between lineages and was not affected by the environment in *A. c. cerana* (Fig. 4B, *A. indica* versus *A. c. cerana* in Bangalore: difference estimate=0.0908, *t*-ratio=0.88, *P*=0.817; *A. indica* versus *A. c. cerana* in Kullu: difference estimate=−0.0199, *t*-ratio=0.19, *P*=0.997; *A. indica* versus *A. c. kashmirensis*: difference estimate=0.1189, *t*-ratio=1.25, *P*=0.623; *A. c. cerana* in Bangalore versus *A. c. cerana* in Kullu: difference estimate=−0.1107, *t*-ratio=−1.07, *P*=0.722; *A. c. cerana* in Bangalore versus *A. c. kashmirensis*: difference estimate=0.0281, *t*-ratio=0.29, *P*=0.989; *A. c. cerana* in Kullu versus *A. c. kashmirensis*: difference estimate=0.1388, *t*-ratio=1.47, *P*=0.516). That is, colonies within lineages had similar levels of individual variation in slopes.

## DISCUSSION

Geographic variation in dance behavior (‘dance dialects’) was proposed very early in studies on honey bee communication (Boch, 1957; von Frisch, 1948), but this idea has not been thoroughly pursued, especially in species other than *A. mellifera*. Our study is the first to compare the slopes of dance calibration curves for multiple colonies of different lineages within *A. cerana*. We found that the two montane lineages *A. c. cerana* and *A. c. kashmirensis* exhibit significantly shallower slopes of the dance–distance calibration curves than the tropical *A. indica*. Furthermore, the difference in the slopes was still present between colonies of *A. c. cerana* and *A. indica* when they were examined at the same experimental site (i.e. Bangalore). However, the slopes of these *A. c. cerana* colonies brought to southern India were also significantly steeper than those of the related colonies tested in the Himalayas. These two results are consistent with the two non-exclusive hypotheses: (i) honey bee populations show variation in dance–distance encoding as an adaptation to changes in foraging ranges due to environmental and social conditions (Dyer and Seeley, 1991; Kohl et al., 2020; Gould, 1982), and (ii) dance–distance encoding is not completely innate (Esch et al., 2001; George et al., 2021; Srinivasan et al., 2000; Tautz et al., 2004). In addition, the finding that the temperate lineages of *A. c.* cerana and *A. c. kashmirensis* show calibration curves with shallower slopes compared with those of the tropical *A. indica* conforms with the differences in slopes reported between temperate and tropical *A. mellifera* populations (Boch, 1957; Gould and Towne, 1987; Schneider, 1989). A possible evolutionary scenario is that shorter flowering seasons and food shortage during the winter in temperate climates might have selected for larger mean foraging ranges and hence shallower slopes of dance–distance curves compared with those of colonies in the tropics (Dyer, 1991; Ruttner, 1988; Seeley and Visscher, 1985).

*Apis c. cerana* colonies tested in Bangalore not only had higher slopes but also showed higher inter-colony variation than colonies of the same population tested in Kullu. These differences may be due to genetic variation or changes in the environment. As flying insects use optical flow to measure flight distances, the generation of dance–distance calibration curves is highly dependent on the visual environment at the experimental site (Esch et al., 2001; Srinivasan et al., 2000; Tautz et al., 2004). So, the difference in the slopes of *A. cerana* colonies tested in Bangalore and Kullu might be a consequence of different optical flow conditions at the different sites. However, an intriguing hypothesis is that the visual optomotor system exhibits some degree of developmental plasticity to facilitate physiological adaptations to variation in visual environments. This could help colonies adapt the dance communication to seasonal variation in flight distances due to changing food availability and foraging ranges. *Apis mellifera* colonies surveyed in the UK demonstrated a 5-fold difference in monthly mean foraging distances ranging from 500 m in spring to 2500 m in autumn (Couvillon et al., 2014, 2015). A recent study on honey bee dance behavior showed that dance–distance encoding involves a period of social learning when the bees start foraging (Dong et al., 2023). This finding indicates that there might be a sensitive period that shapes the dance odometer system. This hypothesis is supported by earlier anatomical studies that demonstrated experience-dependent neuroanatomical plasticity and changes in gene expression in the forager brains during the phase of their orientation flights (Durst et al., 1994; Lutz and Robinson, 2013; Withers et al., 1993). Furthermore, there is also behavioral evidence that the sun compass system shows a phase of behavioral maturation (Dyer and Dickinson, 1994; Lindauer, 1959). In our study, the two colonies in the Himalayas were tested in two consecutive weeks of flowering dearth, whereas the three colonies in Bangalore were tested over a period of 3 months with honey flow indicating a high abundance of food sources (B.K.A.K., personal observations). Detailed studies that test for seasonal changes in calibration curves of individual colonies are needed to substantiate the hypothesis that the odometer system might show developmental plasticity.

Our study also provides some insights regarding individual and colony variation in dance–distance calibration curves. The level of variation among colonies was different among the lineages and changed with the environment. Colonies of the tropical lineage *A. indica* exhibited the greatest variation, and the *A. c. cerana* colonies tested in Bangalore showed a higher level of variation than the one tested in the Himalayas. In contrast, the variation in dance–distance calibration curves among individuals did not differ between lineages. So, tropical lineages did not have greater individual variation than montane lineages. This likely implies that there is a baseline level of variation amongst individuals of a colony. Schürch et al. (2016) examining a total of 75 foragers of three *Apis mellifera mellifera* colonies reported that individual calibrations can vary by as much as 50% independent of environment. The authors suggested that this large variation may be because their colonies were hybrids of different populations. However, it is also possible that this variation is a consequence of the multiple mating of honey bee queens, which generates a high degree of intracolonial genetic diversity (Mattila et al., 2008; Palmer and Oldroyd, 2000; Schürch et al., 2013, 2019). Unfortunately, no one has ever tested variation in dance–distance calibration curves among patrilines. Interestingly, there are reports that full sisters preferentially dance with each other, an adequate mechanism to adjust to the variability in the signal that results from genetic diversity (Duong and Schneider, 2008; Oldroyd et al., 1993).

Finally, combining comparative studies of dance behavior in *A. cerana* and *A. mellifera* with population genomics will be a fruitful approach to identify candidate genes involved in distance communication. Both species evolved in the tropics and later extended their distribution range to temperate climate zones, but this happened on different continents, from Africa to Europe in *A. mellifera* and from South-East Asia to Northern Asia in *A. cerana* (Dogantzis et al., 2021; Ji et al., 2020; Smith, 2020). Thus, the larger foraging ranges and shallower slopes of the dance–distance calibration curves in temperate populations of *A. cerana* and *A. mellifera* represent parallel adaptations that might have evolved in response to similar selection pressures and are based on similar genomic mechanisms (Gallant et al., 2014; Ji et al., 2020; Jones et al., 2023; Pfenning et al., 2014). In the future, our extensive knowledge of honey bee behavior and the growing availability of genome information and molecular tools for all *Apis* species should increase the attractiveness of this group for comparative evolutionary studies on behavioral variation and the underlying molecular and neural mechanisms (Alves et al., 2023; Bastin et al., 2018; Jourjine and Hoekstra, 2021; Kohl et al., 2020; Seeley, 1985; Sen Sarma et al., 2009).

## Supplementary Material

10.1242/jexbio.247510_sup1 Supplementary information

## References

[JEB247510C1] Abrol, D. P. (2013). *Asiatic honeybee Apis cerana*. Dordrecht: Springer.

[JEB247510C2] Ahmad, M. (2023). Winter packings in honey bees (*Apis cerana*) to reduce winter mortality in Kashmir Himalayas. *J. Appl. Entomol.* 3, 21-23.

[JEB247510C3] Alves, D. A., George, E. A., Kaur, R., Brockmann, A., Hrncir, M. and Grüter, C. (2023). Diverse communication strategies in bees as a window into adaptations to an unpredictable world. *Proc. Natl. Acad. Sci. USA* 120, e2219031120. 10.1073/pnas.2219031120 37279263 PMC10268221

[JEB247510C4] Bastin, F., Couto, A., Larcher, V., Phiancharoen, M., Koeniger, G., Koeniger, N. and Sandoz, J.-C. (2018). Marked interspecific differences in the neuroanatomy of the male olfactory system of honey bees (genus *Apis*). *J. Comp. Neurol.* 526, 3020-3034. 10.1002/cne.24513 30417379

[JEB247510C5] Bates, D., Mächler, M., Bolker, B. and Walker, S. (2015). Fitting linear mixed-effects models using {lme4}. *J. Stat. Softw.* 67, 1-48. 10.18637/jss.v067.i01

[JEB247510C6] Beekman, M. and Ratnieks, F. L. W. (2000). Long-range foraging by the honey-bee, *Apis mellifera* L.: honey-bee foraging. *Funct. Ecol.* 14, 490-496. 10.1046/j.1365-2435.2000.00443.x

[JEB247510C7] Boch, R. (1957). Rassenmässige Unterschiede bei den Tänzen der Honigbiene (*Apis mellifica* L.). *Z. Für Vgl. Physiol.* 40, 289-320. 10.1007/BF00340572

[JEB247510C8] Cane, J. (1987). Estimation of bee size using intertegular span (Apoidea). *J. Kansas Entomol. Soc.* 60, 145-147.

[JEB247510C9] Couvillon, M. J., Schürch, R. and Ratnieks, F. L. W. (2014). Waggle dance distances as integrative indicators of seasonal foraging challenges. *PLoS One* 9, e93495. 10.1371/journal.pone.0093495 24695678 PMC3973573

[JEB247510C10] Couvillon, M. J., Riddell Pearce, F. C., Accleton, C., Fensome, K. A., Quah, S. K. L., Taylor, E. L. and Ratnieks, F. L. W. (2015). Honey bee foraging distance depends on month and forage type. *Apidologie* 46, 61-70. 10.1007/s13592-014-0302-5

[JEB247510C11] Dogantzis, K. A., Tiwari, T., Conflitti, I. M., Dey, A., Patch, H. M., Muli, E. M., Garnery, L., Whitfield, C. W., Stolle, E., Alqarni, A. S. et al. (2021). Thrice out of Asia and the adaptive radiation of the western honey bee. *Sci. Adv.* 7, eabj2151. 10.1126/sciadv.abj2151 34860547 PMC8641936

[JEB247510C12] Dong, S., Lin, T., Nieh, J. C. and Tan, K. (2023). Social signal learning of the waggle dance in honey bees. *Science* 379, 1015-1018. 10.1126/science.ade1702 36893231

[JEB247510C13] Duong, N. and Schneider, S. S. (2008). Intra-patriline variability in the performance of the vibration signal and waggle dance in the honey bee, *Apis mellifera*. *Ethology* 114, 646-655. 10.1111/j.1439-0310.2008.01504.x

[JEB247510C14] Durst, C., Eichmüller, S. and Menzel, R. (1994). Development and experience lead to increased volume of subcompartments of the honeybee mushroom body. *Behav. Neural Biol.* 62, 259-263. 10.1016/S0163-1047(05)80025-1 7857249

[JEB247510C15] Dyer, F. (1991). Comparative studies of dance communication: analysis of phylogeny and function. In *Diversity in the Genus Apis* (ed. D. R. Smith), pp. 177-198. CRC Press.

[JEB247510C16] Dyer, F. C. and Dickinson, J. A. (1994). Development of sun compensation by honeybees: how partially experienced bees estimate the sun's course. *Proc. Natl. Acad. Sci. USA* 91, 4471-4474. 10.1073/pnas.91.10.4471 11607474 PMC43807

[JEB247510C17] Dyer, F. C. and Seeley, T. D. (1991). Dance dialects and foraging range in three Asian honey bee species. *Behav. Ecol. Sociobiol.* 28, 227-233. 10.1007/BF00175094

[JEB247510C18] Esch, H. E., Zhang, S., Srinivasan, M. V. and Tautz, J. (2001). Honeybee dances communicate distances measured by optic flow. *Nature* 411, 581-583. 10.1038/35079072 11385571

[JEB247510C19] Gallant, J. R., Traeger, L. L., Volkening, J. D., Moffett, H., Chen, P.-H., Novina, C. D., Phillips, G. N., Anand, R., Wells, G. B., Pinch, M. et al. (2014). Genomic basis for the convergent evolution of electric organs. *Science* 344, 1522-1525. 10.1126/science.1254432 24970089 PMC5541775

[JEB247510C20] George, E. A., Thulasi, N., Kohl, P. L., Suresh, S., Rutschmann, B. and Brockmann, A. (2021). Distance estimation by Asian honey bees in two visually different landscapes. *J. Exp. Biol.* 224, jeb242404. 10.1242/jeb.242404 33795415

[JEB247510C21] Gould, J. L. (1982). Why do honey bees have dialects? *Behav. Ecol. Sociobiol.* 10, 53-56. 10.1007/BF00296395

[JEB247510C22] Gould, J. L. and Towne, W. F. (1987). Evolution of the dance language. *Am. Nat.* 130, 317-338. 10.1086/284713

[JEB247510C23] Grüter, C. and Hayes, L. (2022). Sociality is a key driver of foraging ranges in bees. *Curr. Biol.* 32, 5390-5397.e3. 10.1016/j.cub.2022.10.064 36400034

[JEB247510C24] Hepburn, H. R., Radloff, S. E., Verma, S. and Verma, L. R. (2001). Morphometric analysis of *Apis cerana* populations in the southern Himalayan region. *Apidologie* 32, 435-447. 10.1051/apido:2001142

[JEB247510C64] Hu, Z., Miao, C., Di, N., Zhou, C., Zhang, Y., Yang, J., Xun, L. and Li, Y. (2023). Decoding the dance parameters of eastern honeybee, *Apis cerana*. *Apidologie* 54, 10. 10.1007/s13592-023-00990-5

[JEB247510C25] Ji, Y., Li, X., Ji, T., Tang, J., Qiu, L., Hu, J., Dong, J., Luo, S., Liu, S., Frandsen, P. B. et al. (2020). Gene reuse facilitates rapid radiation and independent adaptation to diverse habitats in the Asian honeybee. *Sci. Adv.* 6, eabd3590. 10.1126/sciadv.abd3590 33355133 PMC11206470

[JEB247510C26] Johnson, R. N., Oldroyd, B. P., Barron, A. B. and Crozier, R. H. (2002). Genetic control of the honey bee (*Apis mellifera*) dance language: segregating dance forms in a backcrossed colony. *J. Hered.* 93, 170-173. 10.1093/jhered/93.3.170 12195031

[JEB247510C27] Jones, B. M., Rubin, B. E. R., Dudchenko, O., Kingwell, C. J., Traniello, I. M., Wang, Z. Y., Kapheim, K. M., Wyman, E. S., Adastra, P. A., Liu, W. et al. (2023). Convergent and complementary selection shaped gains and losses of eusociality in sweat bees. *Nat. Ecol. Evol.* 7, 557-569. 10.1038/s41559-023-02001-3 36941345 PMC11610481

[JEB247510C28] Jourjine, N. and Hoekstra, H. E. (2021). Expanding evolutionary neuroscience: insights from comparing variation in behavior. *Neuron* 109, 1084-1099. 10.1016/j.neuron.2021.02.002 33609484

[JEB247510C29] Klingenberg, C. P. (2011). MorphoJ: an integrated software package for geometric morphometrics. *Mol. Ecol. Resour.* 11, 353-357. 10.1111/j.1755-0998.2010.02924.x 21429143

[JEB247510C30] Kohl, P. L. and Rutschmann, B. (2021). Honey bees communicate distance via non-linear waggle duration functions. *PeerJ* 9, e11187. 10.7717/peerj.11187 33868825 PMC8029670

[JEB247510C31] Kohl, P. L., Thulasi, N., Rutschmann, B., George, E. A., Steffan-Dewenter, I. and Brockmann, A. (2020). Adaptive evolution of honeybee dance dialects. *Proc. R. Soc. B Biol. Sci.* 287, 20200190. 10.1098/rspb.2020.0190 PMC7126084 32126959

[JEB247510C32] Lindauer, M. (1956). Über die Verständigung bei indischen Bienen. *Z. Für Vgl. Physiol.* 38, 521-557. 10.1007/BF00341108

[JEB247510C33] Lindauer, M. (1959). Angeborene und erlernte Komponenten in der Sonnenorientierung der Bienen Bemerkungen und Versuche zu einer Mitteilung von Kalmus. *Z. Für Vgl. Physiol.* 42, 43-62. 10.1007/BF00297689

[JEB247510C34] Lo, N., Gloag, R. S., Anderson, D. L. and Oldroyd, B. P. (2010). A molecular phylogeny of the genus *Apis* suggests that the giant honey bee of the Philippines, *A. breviligula* Maa, and the plains honey bee of southern India, *A. indica* Fabricius, are valid species. *Syst. Entomol.* 35, 226-233. 10.1111/j.1365-3113.2009.00504.x

[JEB247510C35] Lüdecke, D., Ben-Shachar, M. S., Patil, I., Waggoner, P. and Makowski, D. (2021). performance: an R package for assessment, comparison and testing of statistical models. *J. Open Source Softw.* 6, 3139. 10.21105/joss.03139

[JEB247510C36] Lutz, C. C. and Robinson, G. E. (2013). Activity-dependent gene expression in honey bee mushroom bodies in response to orientation flight. *J. Exp. Biol.* 216, 2031-2038. 10.1242/jeb.084905 23678099 PMC3656508

[JEB247510C37] Mattila, H. R., Burke, K. M. and Seeley, T. D. (2008). Genetic diversity within honeybee colonies increases signal production by waggle-dancing foragers. *Proc. R. Soc. B Biol. Sci.* 275, 809-816. 10.1098/rspb.2007.1620 PMC2596908 18198143

[JEB247510C38] Oldroyd, B. P., Rinderer, T. E., Buco, S. M. and Beaman, L. D. (1993). Genetic variance in honey bees for preferred foraging distance. *Anim. Behav.* 45, 323-332. 10.1006/anbe.1993.1037

[JEB247510C39] Oldroyd, B. P., Reddy, M. S., Chapman, N. C., Thompson, G. J. and Beekman, M. (2006). Evidence for reproductive isolation between two colour morphs of cavity nesting honey bees (*Apis*) in south India. *Insectes Soc.* 53, 428-434. 10.1007/s00040-005-0889-2

[JEB247510C40] Otis, G. W. and Smith, D. R. (2021). Drone cell cappings of Asian cavity-nesting honey bees (*Apis* spp.). *Apidologie* 52, 782-791. 10.1007/s13592-021-00864-8

[JEB247510C41] Palmer, K. A. and Oldroyd, B. P. (2000). Evolution of multiple mating in the genus *Apis*. *Apidologie* 31, 235-248. 10.1051/apido:2000119

[JEB247510C42] Pfenning, A. R., Hara, E., Whitney, O., Rivas, M. V., Wang, R., Roulhac, P. L., Howard, J. T., Wirthlin, M., Lovell, P. V., Ganapathy, G. et al. (2014). Convergent transcriptional specializations in the brains of humans and song-learning birds. *Science* 346, 1256846. 10.1126/science.1256846 25504733 PMC4385736

[JEB247510C43] Punchihewa, R. W. K., Koeniger, N., Kevan, P. G. and Gadawski, R. M. (1985). Observations on the dance communication and natural foraging ranges of *Apis cerana*, *Apis dorsata* and *Apis florea* in Sri Lanka. *J. Apic. Res.* 24, 168-175. 10.1080/00218839.1985.11100667

[JEB247510C44] Qiu, L., Dong, J., Li, X., Parey, S. H., Tan, K., Orr, M., Majeed, A., Zhang, X., Luo, S., Zhou, X. et al. (2023). Defining honeybee subspecies in an evolutionary context warrants strategized conservation. *Zool. Res.* 44, 483-493. 10.24272/j.issn.2095-8137.2022.414 36994538 PMC10236295

[JEB247510C45] Riley, J. R., Greggers, U., Smith, A. D., Reynolds, D. R. and Menzel, R. (2005). The flight paths of honeybees recruited by the waggle dance. *Nature* 435, 205-207. 10.1038/nature03526 15889092

[JEB247510C46] Rinderer, T. E. and Beaman, L. D. (1995). Genic control of honey bee dance language dialect. *Theor. Appl. Genet.* 91, 727-732. 10.1007/BF00220950 24169907

[JEB247510C47] Ruttner, F. (1988). *Biogeography and Taxonomy of Honeybees*. Berlin: Springer.

[JEB247510C68] Sasaki, M., Takahashi, H. and Sato, T. (1993). Comparison of the dance dialect and foraging range between *Apis mellifera* and northern most subspecies of *A. cerana* in Japan. *Honeybee Sci.* 14, 49-52.

[JEB247510C48] Schindelin, J., Arganda-Carreras, I., Frise, E., Kaynig, V., Longair, M., Pietzsch, T., Preibisch, S., Rueden, C., Saalfeld, S., Schmid, B. et al. (2012). Fiji: an open-source platform for biological-image analysis. *Nat. Methods* 9, 676-682. 10.1038/nmeth.2019 22743772 PMC3855844

[JEB247510C49] Schneider, S. S. (1989). Spatial foraging patterns of the African honey bee, *Apis mellifera scutellata*. *J. Insect Behav.* 2, 505-521. 10.1007/BF01053351

[JEB247510C50] Schürch, R., Couvillon, M. J., Burns, D. D. R., Tasman, K., Waxman, D. and Ratnieks, F. L. W. (2013). Incorporating variability in honey bee waggle dance decoding improves the mapping of communicated resource locations. *J. Comp. Physiol. A* 199, 1143-1152. 10.1007/s00359-013-0860-4 24132490

[JEB247510C51] Schürch, R., Ratnieks, F. L. W., Samuelson, E. E. W. and Couvillon, M. J. (2016). Dancing to her own beat: honey bee foragers communicate via individually calibrated waggle dances. *J. Exp. Biol.* 219, 1287-1289. 26944504 10.1242/jeb.134874

[JEB247510C52] Schürch, R., Zwirner, K., Yambrick, B. J., Pirault, T., Wilson, J. M. and Couvillon, M. J. (2019). Dismantling Babel: creation of a universal calibration for honey bee waggle dance decoding. *Anim. Behav.* 150, 139-145. 10.1016/j.anbehav.2019.01.016

[JEB247510C65] Seeley, T. D. (1985). *Honeybee Ecology: A Study of Adaptation in Social Life*. Princeton University Press.

[JEB247510C53] Seeley, T. D. and Visscher, P. K. (1985). Survival of honeybees in cold climates: the critical timing of colony growth and reproduction. *Ecol. Entomol.* 10, 81-88. 10.1111/j.1365-2311.1985.tb00537.x

[JEB247510C66] Sen Sarma, M., Rodriguez-Zas, S. L., Hong, F., Zhong, S. and Robinson, G. E. (2009). Transcriptomic profiling of central nervous system regions in three species of honey bee during dance communication behavior. *PLoS One* 4, p.e6408. 10.1371/journal.pone.0006408 PMC2713418 19641619

[JEB247510C54] Smith, D. R. (2020). Biogeography of honey bees. In *Encyclopedia of Social Insects* (ed. C. K. Starr), pp. 1-14. Cham: Springer International Publishing.

[JEB247510C55] Srinivasan, M. V., Zhang, S., Altwein, M. and Tautz, J. (2000). Honeybee navigation: nature and calibration of the “odometer.” *Science* 287, 851-853. 10.1126/science.287.5454.851 10657298

[JEB247510C67] Su, S., Cai, F., Si, A., Zhang, S., Tautz, J. and Chen, S. (2008). East learns from West: Asiatic honeybees can understand dance language of European honeybees. *PLoS One* 3, e2365. 10.1371/journal.pone.0002365 18523550 PMC2391287

[JEB247510C56] Su, Y.-C., Chiu, Y.-F., Warrit, N., Otis, G. W. and Smith, D. R. (2023). Phylogeography and species delimitation of the Asian cavity-nesting honeybees. *Insect Syst. Divers.* 7, 5. 10.1093/isd/ixad015

[JEB247510C57] Tautz, J., Zhang, S., Spaethe, J., Brockmann, A., Si, A. and Srinivasan, M. (2004). Honeybee odometry: performance in varying natural terrain. *PLoS Biol.* 2, e211. 10.1371/journal.pbio.0020211 15252454 PMC449896

[JEB247510C58] Viraktamath, S., Abrol, D. P., Vastrad, A. S., Rajankar, B., Muthuraman, M., Devanesan, S. and Solomon Raju, A. J. (2013). Morphometry of Indian honey bee, *Apis cerana*. Fabricius. In *Monograph on Morphometry and Phylogeography of Honey Bees and Stingless Bees in India* (ed. A. Viraktamath, B. Fakrudin, A. S. Vastrad and S. Mohankumar). Dharwad, India: University of Agricultural Sciences.

[JEB247510C59] von Frisch, K. (1948). Gelöste und ungelöste Rätsel der Bienensprache. *Naturwiss.* 35, 12-23. 10.1007/BF00626624

[JEB247510C60] von Frisch, K. (1967). *The Dance Language and Orientation of Bees*. Cambridge, MA: Belknap Press of Harvard University Press.

[JEB247510C61] Wickham, H. (2016). *ggplot2: Elegant graphics for data analysis*. New York: Springer-Verlag.

[JEB247510C62] Withers, G. S., Fahrbach, S. E. and Robinson, G. E. (1993). Selective neuroanatomical plasticity and division of labour in the honeybee. *Nature* 364, 238-240. 10.1038/364238a0 8321320

[JEB247510C63] Young, A. M., Kohl, P. L., Rutschmann, B., Steffan-Dewenter, I., Brockmann, A. and Dyer, F. C. (2021). Temporal and spatial foraging patterns of three Asian honey bee species in Bangalore, India. *Apidologie* 52, 503-523. 10.1007/s13592-020-00839-1

