# Supplementary material for: Tropical and montane Apis cerana show distinct dance–distance calibration curves
Source: J Exp Biol. 2024 Jul 3;227(13):jeb247510. doi: 10.1242/jeb.247510 (PMC11418176; doi:10.1242/jeb.247510)
Supplement: Supplementary information [file jexbio-227-247510-s1.pdf]

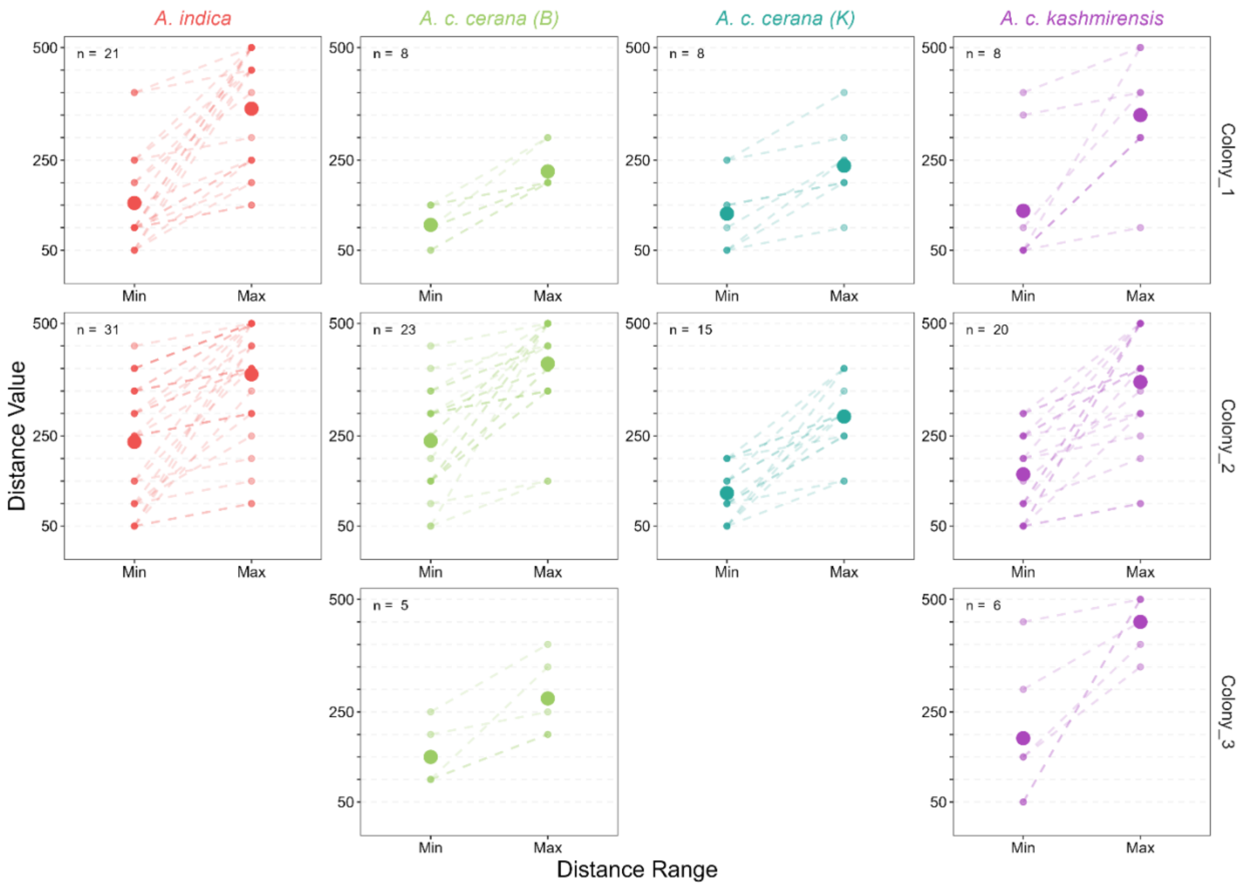

Fig. S1. Variation in distances that individuals were observed dancing. Each subplot represents data of all individuals which were observed dancing at multiple distances from one colony (number of individuals provided as inset text). Smaller circles represent individual values with dashed lines connecting the minimum and maximum distance of the same individual. Larger circles represent the mean values for the colony. Circles are coloured per lineage and location with red for *A. indica*, green for *A. c. cerana* in Bangalore, teal for *A. c. cerana* in Kullu and purple for *A. c. kashmirensis*.

Journal of Experimental Biology • Supplementary information

Table S1. Model comparisons for slopes of *A. cerana* lineages. The table contains information about the Akaike's Information Criterion (AIC), second order AIC (AICc), Bayesian Information Criterion (BIC), Root Mean Squared Error (RMSE), residual standard deviation (Sigma) and relative performance of three models – 2 linear mixed models (LMMs), one with a random effect of colony on intercepts and another with a random effect of bee ID on intercepts and a non-linear mixed model (NLMM) with random effect of colony on the slope equivalent parameter.

Name	AIC	AICc	BIC	RMSE	Sigma	Performance_Score
LMM with colony intercepts as random factor	-336.503	-336.366	-282.632	0.213961	0.214593	0.823527
LMM with bee intercepts as random factor	538.7364	538.7526	613.894	0.237671	0.239908	0.053623
NLMM with colony slopes as random factor	-142.376	-142.211	-83.1177	0.246357	0.024706	0.2

Table S2. Loading values of the morphometric characters on the first three principal components

Measured morphometric characters		PC1	PC2	PC3
Hamuli number		-0.09265	-0.02773	-0.03614
Forewing length (lfw)		0.416517	-0.17188	-0.02776
Forewing width (wfw)		0.423511	-0.16875	0.004622
Length of cubital vein 1 (cub1)		0.35961	0.177534	0.151034
Length of cubital vein 2(cub2)		-0.18272	-0.43663	0.170353
Cubital index (ci)		0.303334	0.418637	-0.07237
Intertegular distance (id)		0.435771	-0.1575	-0.11641
Wing vein angles	A4	-0.01281	-0.41363	0.127886
	B4	0.134947	0.167534	-0.38582
	D7	0.048068	-0.38493	-0.12801
	E9	-0.08611	0.112407	-0.33532
	G18	0.096813	-0.16668	0.17138
	J10	0.109119	0.096257	0.410653
	J16	-0.06391	0.124018	0.38884
	K19	0.353401	-0.20499	0.051394
	L13	0.066794	0.183381	0.143527
	N23	0.07931	0.174898	0.466069
	O26	-0.06962	0.019179	0.208748

Journal of Experimental Biology • Supplementary information

Table S3. Loading values of the individual landmark coordinates on the first three principal components.

Landmarks	PC1	PC2	PC3
x1	0.214446	0.101172	-0.24059
y1	0.166017	-0.045254	0.134636
x2	0.144753	0.154981	-0.185006
y2	0.063313	0.080656	0.049363
x3	-0.02587	0.183241	-0.082152
y3	0.072966	0.077778	0.018151
x4	-0.037993	0.032948	0.088997
y4	-0.007926	0.16212	-0.015276
x5	-0.107637	0.121983	-0.04033
y5	-0.046347	0.218145	-0.163023
x6	-0.288447	-0.0538	-0.079435
y6	-0.124105	0.162497	-0.215755
x7	0.048708	0.126934	0.308514
y7	0.038633	-0.082104	-0.145357
x8	-0.028634	-0.350965	-0.364699
y8	-0.062555	0.158372	-0.016776
x9	0.206558	-0.061054	-0.044675
y9	-0.163996	-0.064216	0.095484
x10	0.26495	-0.00383	0.08109
y10	-0.16121	-0.093888	0.066243
x11	0.15167	-0.15425	-0.105308
y11	-0.150063	0.031403	0.133203
x12	-0.214504	-0.261139	-0.326228
y12	-0.070559	0.173961	-0.067041
x13	-0.472435	-0.104425	-0.102783
y13	-0.004874	0.18273	-0.157173
x14	-0.372604	-0.020914	0.457004
y14	0.070949	0.147968	-0.128871
x15	-0.000963	0.024301	0.130403
y15	-0.035739	0.080502	-0.045898
x16	-0.001946	0.027175	0.144431
y16	0.036282	-0.052994	0.115167
x17	-0.001353	0.070338	0.132833
y17	0.088325	-0.174971	0.072004
x18	0.164213	0.138886	0.083921
y18	0.134449	-0.364382	0.088835
x19	0.212761	-0.093257	0.050461
y19	0.007765	-0.199071	0.092074
x20	0.144327	0.121677	0.093551
y20	0.148676	-0.399251	0.090011

Journal of Experimental Biology • Supplementary information

Table S4. Slope values for individual bees. The table contains information about the estimated slope values of 77 bee IDs and the 95% lower and upper confidence limit (LCL and UCL) around this estimate. The lineages, locations and the colonies individuals belong to are provided along with the starting and maximum feeder distance at which the individual was observing dancing.

Lineage	Location	Colony	Bee ID	Slope [LCL – UCL]	Distance Range
A. indica	Bangalore	1	BAI	0.0044 [0.0037 - 0.0052]	100 - 450
A. indica	Bangalore	1	BA_	0.0037 [0.002 - 0.0054]	200 - 450
A. indica	Bangalore	1	GA	0.0045 [0.0037 - 0.0053]	150 - 500
A. indica	Bangalore	1	GA_	0.0031 [0.0016 - 0.0046]	250 - 450
A. indica	Bangalore	1	GG	0.001 [-0.0022 - 0.0041]	400 - 500
A. indica	Bangalore	1	GT	0.007 [0.0043 - 0.0096]	100 - 250
A. indica	Bangalore	1	RR	0.005 [0.0045 - 0.0056]	50 - 450
A. indica	Bangalore	1	RT	0.0047 [0.0042 - 0.0053]	100 - 500
A. indica	Bangalore	1	RY	0.0048 [0.0042 - 0.0055]	50 - 500
A. indica	Bangalore	1	WB	0.0065 [0.0038 - 0.0092]	150 - 250
A. indica	Bangalore	1	WG	0.0079 [0.0045 - 0.0113]	100 - 200
A. indica	Bangalore	1	WT	0.0043 [0.002 - 0.0066]	200 - 400
A. indica	Bangalore	1	WW	0.0058 [0.0043 - 0.0073]	50 - 250
A. indica	Bangalore	1	YA	0.0045 [0.0041 - 0.0049]	100 - 500
A. indica	Bangalore	1	YY	0.0046 [0.0014 - 0.0077]	100 - 250
A. indica	Bangalore	2	B"	0.003 [0.0018 - 0.0041]	150 - 400
A. indica	Bangalore	2	DBT	0.005 [0.0037 - 0.0064]	300 - 500
A. indica	Bangalore	2	Fg!	0.0049 [0.0023 - 0.0076]	250 - 400
A. indica	Bangalore	2	FgA	0.0058 [0.0026 - 0.009]	300 - 450
A. indica	Bangalore	2	GA	0.0042 [0.003 - 0.0055]	150 - 500

Journal of Experimental Biology • Supplementary information

A. indica	Bangalore	2	GG	0.0044<br>[0.0033 - 0.0055]	100 - 450
A. indica	Bangalore	2	GT	0.005<br>[0.0044 - 0.0057]	50 - 350
A. indica	Bangalore	2	GoT	0.0107<br>[0.0066 - 0.0148]	300 - 400
A. indica	Bangalore	2	PA	0.0024<br>[0.0017 - 0.0031]	50 - 450
A. indica	Bangalore	2	RYT	0.0023<br>[0.0002 - 0.0044]	400 - 500
A. indica	Bangalore	2	VT	0.003<br>[0.0015 - 0.0046]	250 - 500
A. indica	Bangalore	2	Y.	0.0017<br>[-0.0002 - 0.0037]	350 - 500
A. indica	Bangalore	2	YY	0.0043<br>[0.0023 - 0.0063]	400 - 500
A. c. cerana	Bangalore	1	BAI	0.0032<br>[0.0015 - 0.0048]	50 - 200
A. c. cerana	Bangalore	1	GT	0.0043<br>[0.0001 - 0.0085]	100 - 200
A. c. cerana	Bangalore	1	OA	0.0058<br>[0.0037 - 0.008]	150 - 300
A. c. cerana	Bangalore	1	RG	0.0027<br>[0.0006 - 0.0047]	50 - 200
A. c. cerana	Bangalore	1	RT	0.002<br>[-0.0021 - 0.0061]	100 - 200
A. c. cerana	Bangalore	1	RW	0.004<br>[0.0024 - 0.0055]	100 - 300
A. c. cerana	Bangalore	2	BB	0.0018<br>[0.0006 - 0.0031]	250 - 500
A. c. cerana	Bangalore	2	BT	0.0021<br>[0.0014 - 0.0028]	150 - 500
A. c. cerana	Bangalore	2	RA	0.002<br>[0.0015 - 0.0025]	150 - 500
A. c. cerana	Bangalore	2	RT	0.0026<br>[0.0014 - 0.0037]	150 - 400
A. c. cerana	Bangalore	2	RW	0.0026<br>[0.0011 - 0.0041]	150 - 350
A. c. cerana	Bangalore	2	RWA	0.0019<br>[-0.0004 - 0.0041]	300 - 450
A. c. cerana	Bangalore	2	WA	0.0027<br>[0.0016 - 0.0037]	150 - 400

Journal of Experimental Biology • Supplementary information

A. c. cerana	Bangalore	2	WR	0.0004 [-0.0013 - 0.0022]	250 - 500
A. c. cerana	Bangalore	2	YA	0.0022 [0.001 - 0.0034]	150 - 350
A. c. cerana	Bangalore	2	YT	0.0016 [0.001 - 0.0023]	50 - 500
A. c. cerana	Bangalore	2	YYG	0.0019 [0.001 - 0.0028]	200 - 500
A. c. cerana	Bangalore	3	GP	0.0028 [0.0005 - 0.005]	250 - 400
A. c. cerana	Kullu	1	OT	0.0009 [-0.0008 - 0.0026]	250 - 400
A. c. cerana	Kullu	1	P*	0.0025 [-0.0002 - 0.0052]	100 - 250
A. c. cerana	Kullu	1	WT	0.0017 [-0.0005 - 0.0039]	50 - 250
A. c. cerana	Kullu	2	BRT	0.0021 [0.0008 - 0.0034]	100 - 300
A. c. cerana	Kullu	2	DBT	0.0012 [-0.0018 - 0.0043]	150 - 300
A. c. cerana	Kullu	2	GRT	0.0007 [-0.0021 - 0.0036]	150 - 300
A. c. cerana	Kullu	2	GT	0.0014 [-0.0036 - 0.0063]	50 - 150
A. c. cerana	Kullu	2	LBT	0.0019 [0.0009 - 0.0028]	50 - 400
A. c. cerana	Kullu	2	LGT	0.0016 [0.0004 - 0.0028]	50 - 300
A. c. cerana	Kullu	2	LGT_	0.003 [0.0016 - 0.0045]	200 - 400
A. c. cerana	Kullu	2	RA	0.0033 [0.0006 - 0.006]	100 - 250
A. c. cerana	Kullu	2	RBT	0.0018 [-0.0002 - 0.0038]	100 - 250
A. c. cerana	Kullu	2	WRT	0.0012 [-0.0004 - 0.0027]	150 - 400
A. c. cerana	Kullu	2	WT	0.0028 [0.0001 - 0.0054]	200 - 300
A. c. kashmirensis	Kashmir	1	G/	0.0017 [0.0008 - 0.0026]	100 - 400
A. c. kashmirensis	Kashmir	1	PT	0.0017 [0.0007 - 0.0027]	50 - 300

Journal of Experimental Biology • Supplementary information

A. c. kashmirensis	Kashmir	1	WA	0.0022<br>[0.0013 - 0.0032]	50 - 300
A. c. kashmirensis	Kashmir	1	YA	0.0041<br>[0.0023 - 0.0059]	50 - 300
A. c. kashmirensis	Kashmir	2	BA	0.0022<br>[-0.0001 - 0.0045]	250 - 400
A. c. kashmirensis	Kashmir	2	BB	0.0027<br>[0.0014 - 0.0039]	250 - 500
A. c. kashmirensis	Kashmir	2	BT	0.0033<br>[0.0023 - 0.0043]	50 - 300
A. c. kashmirensis	Kashmir	2	FGI	0.0025<br>[0.0007 - 0.0044]	150 - 350
A. c. kashmirensis	Kashmir	2	OA	0.0019<br>[0.0005 - 0.0032]	200 - 500
A. c. kashmirensis	Kashmir	2	PP	0.0025<br>[0.0002 - 0.0049]	250 - 400
A. c. kashmirensis	Kashmir	2	RWT	0.0013<br>[0.0004 - 0.0023]	50 - 500
A. c. kashmirensis	Kashmir	2	WRT	0.0023<br>[0.0011 - 0.0034]	100 - 500
A. c. kashmirensis	Kashmir	2	YT	0.0018<br>[0.0009 - 0.0027]	100 - 500
A. c. kashmirensis	Kashmir	3	FG_	0.0018<br>[0.0009 - 0.0028]	50 - 500
A. c. kashmirensis	Kashmir	3	GWT	0.0011<br>[-0.0005 - 0.0027]	300 - 450
A. c. kashmirensis	Kashmir	3	WT	0.0019<br>[0.0012 - 0.0026]	50 - 500
A. c. kashmirensis	Kashmir	3	WW	0.0018<br>[-0.0002 - 0.0037]	150 - 400

Journal of Experimental Biology • Supplementary information
